# Breaking the silence on drug-facilitated sexual assault: disclosure patterns and barriers to reporting among young people in Spain

**DOI:** 10.3389/fpubh.2026.1924643

**Published:** 2026-10-19

**Authors:** Pablo Prego-Meleiro, Luis Sordo, Paula Meira-Pliego, José Pulido, Gemma Montalvo

**Affiliations:** 1 Department of Public Health and Maternal and Child Health, Faculty of Medicine, Universidad Complutense de Madrid, Madrid, Spain; 2 Centro de Investigación Biomédica en Red de Epidemiología y Salud Pública (CIBERESP), Madrid, Spain; 3 Instituto Universitario de Investigación en Ciencias Policiales, Universidad de Alcalá, Alcalá de Henares, Spain; 4 Observatorio Universitario de Violencia Sexual Facilitada por Drogas, Universidad de Alcalá, Alcalá de Henares, Spain; 5 Fundación para la Investigación Biomédica del Hospital Clínico San Carlos (IDISSC), Madrid, Spain; 6 Department of Analytical Chemistry, Physical Chemistry and Chemical Engineering, Universidad de Alcalá, Alcalá de Henares, Madrid, Spain

**Keywords:** disclosure, drug-facilitated sexual assault, help-seeking, nightlife, prevention, sexual violence, underreporting, victimization

## Abstract

**Introduction:**

Drug-facilitated sexual assault (DFSA) is a common form of sexual violence among young people in party settings, predominantly involving opportunistic assaults facilitated by alcohol. Although highly prevalent, little is known about disclosure patterns following DFSA, despite their implications for prevention and survivors' recovery. This study aimed to estimate the prevalence of disclosure of DFSA experiences in Spain, with a particular focus on opportunistic assaults, and to identify victim and assault characteristics associated with disclosure.

**Materials and methods:**

A cross-sectional study was conducted using an anonymous online survey administered to a national sample of young adults aged 18–35 years residing in Spain. Analyses were restricted to participants reporting lifetime DFSA victimization in party settings. Disclosure was classified as no disclosure, disclosure to the close social environment only, or disclosure to authorities. The prevalence of disclosure and its associations with sociodemographic and assault characteristics were examined using generalized ordinal logistic regression and partial proportional odds models.

**Results:**

Among victims of DFSA in party settings, 17.5% had never disclosed the assault, 78.2% disclosed it only to their close social environment, and only 4.3% disclosed it to authorities. Women showed higher odds of disclosure than men. Perpetrator strategy showed the strongest association with disclosure to authorities, although estimates were imprecise because formal disclosure was uncommon. Opportunistic DFSA involving voluntary substance consumption showed particularly low levels of formal disclosure.

**Discussion:**

Most victims of DFSA disclose the assault to someone but do not reach formal support systems. This gap may compromise secondary prevention by limiting timely access to healthcare, while also hindering primary prevention by perpetuating the invisibility of DFSA and restricting surveillance and public awareness. Perpetrator strategy emerged as the factor most strongly associated with disclosure, suggesting that opportunistic DFSA remains particularly hidden. To inform prevention efforts, we propose a conceptual model in which an interconnected set of barriers may hinder victims' disclosure and access to support. These findings highlight the need for prevention policies and awareness campaigns that explicitly address opportunistic DFSA rather than portraying DFSA primarily as covert drink spiking, while also strengthening victim-support interventions aimed at improving recognition of opportunistic DFSA and reducing barriers to formal help-seeking.

## 1 Introduction

Sexual violence constitutes a major public health challenge worldwide (1). According to the World Health Organization, it encompasses a wide range of non-consensual sexual behaviors that violate an individual's sexual autonomy (2). Its consequences extend across a broad spectrum of adverse physical, mental, and social outcomes, generating a substantial burden for individuals, communities, and health systems (3). In Spain, sexual violence has gained increasing social and legal visibility in recent years (4). The Organic Law 10/2022 on the Comprehensive Guarantee of Sexual Freedom placed consent at the core of sexual-offense legislation and explicitly recognized sexual acts involving exploitation of a victim's vulnerability or incapacity as sexual assault (5). This legal development has occurred within a broader social context in which nightlife is an important setting for sexual violence among young people and where alcohol and other substance use may intersect with sexual interactions, consent, and gendered perceptions of responsibility (6, 7).

Among the different forms of sexual violence, drug-facilitated sexual assault (DFSA) occupies a particularly relevant place in youth party settings (8). DFSA refers to sexual assaults in which perpetrators exploit a victim's reduced capacity to resist, understand, or consent to sexual activity because of the effects of alcohol, other drugs, or psychoactive medications. In the DFSA literature, two main perpetrator strategies are commonly distinguished: opportunistic DFSA, in which the perpetrator exploits intoxication resulting from the victim's voluntary substance use, and proactive DFSA, in which the perpetrator deliberately causes or increases intoxication through covert administration, force, or coercion (8, 9). These terms refer exclusively to the perpetrator's modus operandi and do not imply any responsibility on the part of victims who voluntarily consumed alcohol or other substances. In Spain, approximately one in two women and one in four men aged 18–35 years have experienced DFSA at least once in their lifetime while partying (10). This sex disparity should be understood within broader gendered dynamics surrounding sexual violence in Spanish nightlife contexts: women report lower perceived safety and greater victim-blaming related to substance use, whereas men have shown greater acceptance of sexual activity with individuals unable to express consent because of drugs and greater attribution of sexual assault to victims' alcohol or drug use (6, 11). These findings may help contextualize the disproportionate burden observed among women, while also underscoring that DFSA victimization among men is substantial.

International evidence indicates that most survivors of sexual assault eventually disclose their experience to someone (12), although disclosure occurs primarily within informal support networks, such as friends, family members, or intimate partners (13). By contrast, disclosure to formal support providers—including police, healthcare professionals, mental health professionals, or rape crisis workers—is substantially less frequent (12, 14, 15). A recent review reported sexual assault disclosure rates ranging from 41% to 100% to informal sources, compared with only 0% to 16% to formal sources (16). However, these findings derive primarily from studies conducted outside Spain. Evidence on sexual assault disclosure patterns in the Spanish context remains limited, particularly for DFSA and for victims identified outside healthcare, forensic, or judicial settings.

Beyond estimating disclosure rates, identifying the barriers that inhibit disclosure is essential because remaining silent may delay access to healthcare, psychological support, legal protection, and other forms of assistance. Sexual assault survivors may face multiple, interrelated barriers to disclosure, including shame, guilt and self-blame; fear of being disbelieved or blamed by others; concerns about retaliation or negative social consequences; uncertainty about whether the experience qualifies as sexual assault; and distrust or anticipated negative responses from formal institutions (13–16). Disclosure is also shaped by the reactions received from others: unsupportive responses from relatives, peers, professionals, or institutions may reinforce self-blame, undermine confidence in the legitimacy of the experience, and discourage subsequent help-seeking (17, 18). Similar processes have been described across different stages of the course of life. Research on childhood and adolescent sexual abuse identifies shame, guilt, self-blame, anticipated negative consequences, limited support, and characteristics of the victim–perpetrator relationship as important barriers to disclosure (19, 20). These findings underscore that disclosure is not a single decision but a socially embedded process that may determine whether victims gain access to protection and recovery resources.

These issues are particularly important for DFSA research in Spain. Much of the available Spanish evidence has been derived from healthcare or forensic samples, an approach that necessarily captures victims who have reached formal systems and therefore inherently excludes victims who never disclose the assault to authorities or seek professional assistance (21, 22). Consequently, little is known about disclosure patterns among DFSA victims, including the factors that distinguish non-disclosure, disclosure to informal networks, and disclosure to formal authorities. Addressing this gap is necessary to estimate the hidden burden of DFSA more accurately and to understand which characteristics of victims and assaults are associated with silence, informal and formal disclosure.

Based on previous research, we hypothesized that: (1) disclosure of DFSA would occur predominantly within informal social networks, whereas disclosure to formal authorities would be uncommon; (2) disclosure patterns would differ by sex, with women showing a greater likelihood of disclosure than men; and (3) assault characteristics that more closely conform to socially recognizable representations of sexual violence, including greater invasiveness, multiple perpetrators, and involuntary substance administration, would be associated with a greater likelihood of formal disclosure. Accordingly, this study aimed to estimate the prevalence and patterns of disclosure among young people who had experienced DFSA in nightlife settings in Spain and to quantify the hidden burden of cases that remained undisclosed. We additionally examined sociodemographic, assault-related, and perpetrator-strategy characteristics associated with non-disclosure, informal disclosure and disclosure to formal authorities.

## 2 Materials and methods

### 2.1 Study design and participants

A cross-sectional study was conducted using an anonymous, self-administered online questionnaire delivered through a computer-assisted web interviewing (CAWI) platform. The target population comprised individuals aged 18–35 years residing in Spain, representing 9,250,779 people in 2022, of whom 50.85% were men (23). Based on a 95% confidence level, a 2.5% margin of error, and a conservative expected prevalence of 50%, a minimum sample size of 1,537 participants was estimated following standard statistical recommendations (24).

Participants were recruited through a certified research company operating an online panel of more than 100,000 registered members in Spain. The company complies with ISO 20252 standards and maintains verified participant profiles for social and health research purposes. Panel members are recruited through paid online strategies and undergo continuous profile validation and updating procedures.

To obtain a sample reflecting the demographic structure of the Spanish population, quota sampling was implemented according to sex, age group (18–24 and 25–35 years), and region of residence (17 autonomous communities). Potential participants were selected using an automated routing system designed to reduce self-selection bias and were contacted through personalized email invitations and mobile notifications. Recruitment quotas were continuously monitored and adjusted according to response and attrition rates.

The survey instrument was developed by an interdisciplinary research team and drew upon established sources, including the Sexual Experiences Survey–Short Form Victimization (25) and the 2019 Macro-Survey on Violence Against Women (26). Prior to fieldwork, the questionnaire underwent pilot testing, including cognitive debriefing with more than 100 participants, to evaluate comprehension, clarity, and content validity.

Several procedures were implemented to ensure data quality. Access to the survey required captcha verification and duplicate-response checks, while consistency controls included validation of self-reported age and sex, attention-check items, minimum completion-time thresholds, and mandatory responses. No identifying information was collected, including IP addresses or cookies, in order to preserve participant anonymity and reduce social desirability bias.

Of the 1,707 individuals invited to participate, 106 (6.2%) either declined participation or did not complete the questionnaire, resulting in a final analytical sample of 1,601 respondents. Among them, 36.4% (*n* = 583) reported having experienced DFSA while partying, constituting the study population analyzed in the present study. Of the 583 participants included in the present study, the mean age was approximately 27 years (SD 5). Among those reporting their sex, 374 (64.6%) were women and 205 (35.4%) were men; four participants selected “prefer not to answer.” Among participants with available educational data, 317 (54.7%) had university-level education and 263 (45.3%) had non-university education.

### 2.2 Variables

One variable assessed disclosure of the assault, reflecting increasing levels of formalization in disclosure behaviors. It was defined as “Thinking about the most recent time this happened, who did you tell about what happened?” [“no disclosure,” “disclosure to the close social environment only,” or “disclosure to authorities (with or without disclosure to the close social environment)”]. Close social environment included friends, relatives, current or former partners, educational staff, work-related contacts, helplines for victims, social media, lawyers, and other informal recipients. Authorities included healthcare or psychological services and police or judicial institutions.

DFSA victimization while partying was assessed as “having ever experienced alcohol- and/or other drug-facilitated sexual assault during or just after partying” (no/yes). Sexual acts included unwanted sexual touching, kissing, masturbation by another person, oral sex, or penetration. All participants reporting DFSA victimization were classified according to the assault invasiveness: less invasive (experiencing unwanted touching and/or kissing, exclusively) and more invasive (experiencing unwanted masturbation by another person and/or oral sex and/or penetration [vaginal and/or anal], with or without touching and/or kissing).

Additional independent variables included age, sex, educational attainment (university; non-university), sexual orientation (heterosexual; sexual minority), political ideology (left; right), nationality (Spanish only; other/dual nationality), and socioeconomic status. Sex was assessed as recorded on the participant's national identity card, passport, or equivalent official identification document, with response options “man,” “woman,” and “prefer not to answer.” Political ideology was originally assessed on a 10-point scale and subsequently dichotomized into left (1–5) and right (6–10). Socioeconomic status was measured through monthly household net income and categorized as low to low/medium ( ≤ €2,000/month) or medium/high to high (>€2,000/month).

All variables describing the characteristics of the DFSA experience referred to the most recent victimization episode. Accordingly, participants who had experienced multiple DFSA incidents provided information only in relation to their most recent assault. These variables were based on victims' statements and included perpetrator sex (man; woman), number of perpetrators (one; multiple), previous relationship with the perpetrator (known; unknown), assault strategy, and assault invasiveness. Assault strategy was classified as opportunistic DFSA involving the victim's voluntary alcohol consumption only; opportunistic DFSA involving the victim's voluntary consumption of other drugs, with or without alcohol (cannabis, stimulants, hallucinogens, new psychoactive substances, or pharmaceutical drugs); and proactive DFSA involving the victim's involuntary consumption of substances, including covert administration, injection, force, or coercion. Substances associated with proactive DFSA included those commonly referred to as “rape drugs,” such as GHB, ketamine, or scopolamine (*burundanga*). An expanded extract of the questionnaire, including the items relevant to the variables used in the present study, is provided in Multimedia Appendix 1.

### 2.3 Statistical analysis

#### 2.3.1 Descriptive, bivariate and multivariable analyses

Analyses were organized according to the study hypotheses. To address the first hypothesis, concerning the predominance of informal over formal disclosure, we estimated the prevalence of the three disclosure categories overall. To address the second hypothesis, concerning sex differences in disclosure, sex-specific prevalence estimates were calculated and the association between sex and disclosure was further examined in the multivariable models. To address the third hypothesis, concerning assault characteristics associated with formal disclosure, associations between disclosure patterns and assault-related variables were examined in bivariate and multivariable analyses. Bivariate associations were assessed using Pearson's chi-square test or Fisher's exact test for categorical variables and analysis of variance (ANOVA) for continuous variables.

Generalized ordinal logistic regression and partial proportional odds models (27) were used to examine the association between DFSA disclosure patterns (“no disclosure,” “disclosure to the close social environment only,” or “disclosure to authorities”) and sociodemographic and assault-related characteristics. This approach was selected because the outcome represented an ordinal process with potentially non-proportional effects across thresholds. Specifically, it allowed the simultaneous modeling of two related but distinct processes: (1) disclosure vs. non-disclosure and (2) disclosure to authorities vs. disclosure only within the close social environment or non-disclosure. Because the proportional odds assumption was not fully met for all covariates, partial proportional odds models were estimated when appropriate. Likelihood ratio tests were used to compare fully generalized ordered logit models with partial proportional odds specifications, retaining the most parsimonious model that adequately fitted the data (28). The proportional odds assumption for the final models was evaluated using Wald tests.

Variables were selected for the multivariable models based on theoretical relevance and bivariate associations. Sex, age, and educational level were retained as sociodemographic covariates, while assault- and perpetrator-related variables showing *P* < 0.05 in bivariate analyses were considered for sequential inclusion. A hierarchical modeling strategy was applied through the sequential inclusion of sociodemographic and assault-related variables to assess the extent to which associations with disclosure patterns persisted after adjustment for progressively more proximal characteristics of the victimization experience. Model 1 adjusted for victims' sociodemographic characteristics (sex, age, and educational level) and assault invasiveness. Model 2 additionally included perpetrator-related variables (“number of perpetrators” and “previous relationship with the perpetrator”). Model 3 further incorporated “perpetrator strategy.” This progressive adjustment through increasingly complex models allowed the assessment of whether associations between disclosure patterns and specific assault characteristics persisted after accounting for other characteristics of the victimization experience. Likelihood-ratio tests supported the use of partially proportional generalized ordinal logistic regression models over fully generalized models. Violations of the proportional odds assumption were mainly observed for variables related to assault invasiveness and perpetrator strategy. The final model included 429 participants due to missing information on assault-related characteristics, particularly perpetrator strategy.

Odds ratios (ORs) and corresponding 95% confidence intervals (95% CIs) were reported. All analyses were performed using Stata version 19 (StataCorp LLC, College Station, TX, USA).

#### 2.3.2 Sensitivity and supplementary analyses

Given the small number of disclosures to authorities in some perpetrator-strategy categories, a sensitivity analysis was conducted to assess the potential influence of sparse-data bias. For this analysis, disclosure was dichotomized as disclosure to authorities vs. no disclosure to authorities, corresponding to the upper cumulative contrast of the generalized ordered logistic model. The same analytic sample and covariates as in Model 3 were used. Conventional binary logistic regression was first estimated, followed by Firth's penalized logistic regression to reduce small-sample and separation-related bias.

Because perpetrator strategy had the highest proportion of missing data, additional analyses were conducted to assess whether missingness in this variable was associated with disclosure status or other observed characteristics. Missingness in perpetrator strategy was first compared across the three disclosure categories and between any disclosure vs. no disclosure using chi-square tests. Participants with observed vs. missing perpetrator strategy data were also compared according to sex, age, educational level, assault invasiveness, number of perpetrators, and victim–perpetrator relationship. Finally, logistic regression models were fitted with missingness in perpetrator strategy as the dependent variable and disclosure status as the main explanatory variable, adjusting for the same observed sociodemographic and assault-related covariates.

Because assault invasiveness and perpetrator strategy could plausibly be related, their association was examined using a chi-square test and Cramér's V, and variance inflation factors were calculated as a diagnostic for multicollinearity.

### 2.4 Ethical considerations

The study protocol was approved by the Research Ethics Committee of the University of Alcalá (CEIP2022/2/040). All participants provided informed consent before accessing the questionnaire. Participation was entirely voluntary. To protect confidentiality, the survey was fully anonymous and did not collect personal identifiers. Neither IP addresses nor cookies were recorded, and all analyses were conducted using aggregated data, preventing the identification of individual participants.

## 3 Results

### 3.1 Descriptive and bivariate analysis

Among individuals who experienced DFSA in party settings, 82.5% (*n* = 481) reported some form of disclosure, predominantly to their close social environment (78.2%; *n* = 456). Only 4.3% (*n* = 25) disclosed the assault to authorities. Among these 25 participants, 18 disclosed to healthcare or psychological services and 11 to police or judicial institutions. These corresponded to 3.1% and 1.9% of the 583 participants included in the disclosure analysis and to 72.0% and 44.0% of those who disclosed to authorities, respectively. Four participants disclosed to both types of formal services (0.7% of the disclosure-analysis sample; 16.0% of those who disclosed to authorities). Women showed a higher prevalence of any disclosure than men (86.4% vs. 76.6%; *p* = 0.003; Table 1).

**Table 1:** Prevalence of disclosure of DFSA experiences in partying settings, according to victims' self-reports.

Disclosure category	Disclosure of DFSA experiences						
	Total		Women^†^		Men^†^		***P***
	***N*** **(%)**	**95% CI**	***N*** **(%)**	**95% CI**	***N*** **(%)**	**95% CI**	
Any disclosure	481 (82.5)	79.2–85.4	323 (86.4)	82.5–89.5	157 (76.6)	70.3–81.9	0.003
Disclosure to close social environment only	456 (78.2)	74.7–81.4	309 (82.6)	78.4–86.1	146 (71.2)	64.6–77.0	
Disclosure to authorities ^a^	25 (4.3)	2.9–6.3	14 (3.7)	2.2–6.2	11 (5.4)	3.0–9.5	
No disclosure	102 (17.5)	14.6–20.8	51 (13.6)	10.5–17.5	48 (23.4)	18.1–29.7	

^a^Disclosure to healthcare, psychological, police, or judicial services, regardless of whether the assault was also disclosed to members of the close social environment.

^†^Overall estimates include four participants who selected ‘prefer not to answer' for sex.

Women were more likely than men to disclose to their close social environment (82.6% vs. 71.2%) but less likely to disclose to authorities (3.7% vs. 5.4%; *p* = 0.006). Similarly, higher educational attainment was associated with slightly higher disclosure to the close social environment (78.9% vs. 77.6%) but lower disclosure to authorities (2.5% vs. 6.5%; *p* = 0.055). No significant differences were observed according to sexual orientation, political ideology, nationality, or socioeconomic status (Table 2).

**Table 2:** Bivariate associations between DFSA disclosure patterns and victim characteristics.

**Victim characteristics**	DFSA disclosure pattern, ***N*** (%)			
	Yes		**No**	
	**Social environment** ^a^	**Authorities** ^b^		***P***
No of cases	456	25	102	
**Sex**				0.006
Man^†^	146 (71.2)	11 (5.4)	48 (23.4)	
Woman^†^	309 (82.6)	14 (3.7)	51 (13.6)	
Age (years)				0.798
Mean ± SD	27 ± 5	28 ± 4	27 ± 5	
Sexual orientation				0.142
Heterosexual	333 (79.3)	14 (3.3)	73 (17.4)	
Sexual minority	117 (75.5)	11 (7.1)	27 (17.4)	
Political ideology				0.307
Left-wing	358 (79.4)	20 (4.4)	73 (16.2)	
Right-wing	98 (74.2)	5 (3.8)	29 (22.0)	
Nationality				0.115
Spanish nationality only	407 (79.7)	19 (3.7)	85 (16.6)	
Other or dual nationality	44 (69.8)	5 (7.9)	14 (22.2)	
Socioeconomic level				0.211
Low to medium low	210 (79.9)	15 (5.7)	38 (14.5)	
Medium high to high	168 (77.8)	7 (3.2)	41 (19.0)	
Educational level				0.055
Non-university	204 (77.6)	17 (6.5)	42 (16.0)	
University	250 (78.9)	8 (2.5)	59 (18.6)	

^a^Disclosure of the DFSA experience to members of the close social environment only.

^b^Disclosure of the DFSA experience to authorities (judicial, police, and/or healthcare services) regardless of whether the assault was also disclosed to members of the close social environment.

^†^Overall estimates include four participants who did not report their sex.

Regarding assault severity, more invasive assaults were associated with lower disclosure to the close social environment than less invasive assaults (65.4% vs. 81.0%) but higher disclosure to authorities (13.5% vs. 2.3%; *p* < 0.001). Regarding perpetrator strategy, disclosure to the close social environment was more frequent in opportunistic assaults than in proactive assaults (79.7% and 73.4% vs. 58.5%). By contrast, proactive assaults were characterized by substantially higher disclosure to authorities than opportunistic assaults, especially those involving alcohol only (34.2% vs. 4.1% and 1.4%; *p* < 0.001). With respect to the number of perpetrators, disclosure was more frequent in assaults involving more than one perpetrator than in those committed by a single perpetrator, slightly so for disclosure to the close social environment (79.4% vs. 78.9%) and much more clearly for disclosure to authorities (14.7% vs. 3.2%; *p* = 0.004). Regarding the previous relationship between victim and perpetrator, assaults perpetrated by someone known to the victim showed lower disclosure to the close social environment than assaults perpetrated by unknown perpetrators (73.7% vs. 82.0%) but higher disclosure to authorities (6.4% vs. 3.0%; *p* = 0.035). No significant differences were observed according to perpetrator sex (Table 3).

**Table 3:** Bivariate associations between DFSA disclosure patterns and assault-related characteristics.

Assault-related characteristics	DFSA disclosure pattern, ***N*** (%)			
	Yes		**No**	
	**Social environment** ^a^	**Authorities** ^b^		***P***
**Invasiveness of the assault**				< 0.001
Less invasive^c^	388 (81.0)	11 (2.3)	80 (16.7)	
More invasive^d^	68 (65.4)	14 (13.5)	22 (21.2)	
Perpetrator strategy				< 0.001
Opportunistic DFSA (alcohol only)	291 (79.7)	5 (1.4)	69 (18.9)	
Opportunistic DFSA (other drugs ± alcohol)	36 (73.4)	2 (4.1)	11 (22.5)	
Proactive DFSA (involuntary substance consumption) ^e^	24 (58.5)	14 (34.2)	3 (7.3)	
Number of perpetrators				0.004
One perpetrator	415 (78.9)	17 (3.2)	94 (17.9)	
Multiple perpetrators	27 (79.4)	5 (14.7)	2 (5.9)	
Previous relationship between victim and perpetrator				0.035
Known	174 (73.7)	15 (6.4)	47 (19.9)	
Unknown	273 (82.0)	10 (3.0)	50 (15.0)	
Perpetrator sex				0.123
Woman	95 (73.1)	5 (3.9)	30 (23.1)	
Man	348 (80.6)	18 (4.2)	66 (15.3)	

^a^Disclosure of the DFSA experience to members of the close social environment only.

^b^Disclosure of the DFSA experience to authorities (judicial, police, and/or healthcare services) regardless of whether the assault was also disclosed to members of the close social environment.

^c^Less invasive assault: unwanted touching and/or kissing exclusively.

^d^More invasive assault: unwanted masturbation by another person, oral sex, and/or vaginal or anal penetration, with or without unwanted touching and/or kissing.

^e^Proactive DFSA included involuntary substance consumption through covert administration, force, coercion, or injection.

Data are based on valid responses from participants who reported having experienced DFSA while partying. Percentages were calculated excluding missing values.

### 3.2 Multivariable analyses

Associations with DFSA disclosure patterns changed across successive models as additional victim- and assault-related characteristics were introduced (Table 4). Model 3 was estimated using 429 participants with complete data for all included covariates, with the reduction in sample size largely reflecting missing information on perpetrator strategy.

**Table 4:** Generalized ordinal logistic regression models of DFSA disclosure patterns according to victim characteristics, assault characteristics, and perpetrator strategy.

Predictor variables	DFSA disclosure pattern					
	M1 (*n* = 576)		M2 (*n* = 546)		M3 (*n* = 429)	
	Victim characteristics + Assault invasiveness		M1 + Perpetrator characteristics		M2 + Perpetrator strategy	
	**Any disclosure vs. no disclosure**	**Authorities vs. environment/ no disclosure** ^†^	**Any disclosure vs. no disclosure**	**Authorities vs.environment/no disclosure** ^†^	**Any disclosure vs no disclosure**	**Authorities vs.environment/no disclosure** ^†^
	**OR**^a^ **(95% CI)**	**OR**^a^ **(95% CI)**	**OR**^a^ **(95% CI)**	**OR**^a^ **(95% CI)**	**OR**^a^ **(95% CI)**	**OR**^a^ **(95% CI)**
**Sex**						
Man	Ref.	Idem^*^	Ref.	Idem^*^	Ref.	Idem^*^
Woman	1.80 (1.17–2.76)^*^		1.83 (1.17–2.87) ^*^		1.74 (1.06–2.87) ^*^	
Age (years)	1.02 (0.97–1.06)	Idem	1.01 (0.96–1.05)	Idem	0.97 (0.93 – 1.02)	Idem
Educational level						
Non-university	Ref.	Idem	Ref.	Idem	Ref.	Idem
University	0.69 (0.45–1.06)		0.69 (0.44–1.08)		0.69 (0.42–1.14)	
Invasiveness of the assault						
Less invasive^b^	Ref.	Ref.	Ref.	Ref.	Ref.	Idem
More invasive^c^	0.75 (0.44–1.30)	7.27 (3.18–16.65)^**^	0.62 (0.35–1.12)	5.55 (2.22–13.89)^**^	0.52 (0.27 – 1.00)	
Number of perpetrators						
One perpetrator	-	-	Ref.	Idem^*^	Ref.	Idem
Multiple perpetrators			3.34 (1.32–8.46)^*^		2.57 (0.87–7.62)	
Previous relationship between victim and perpetrator						
Known	-	-	Ref.	Idem	Ref.	Idem
Unknown			1.12 (0.71–1.75)		1.11 (0.67–1.84)	
**Perpetrator strategy**						
Opportunistic DFSA (alcohol only)	-	-	-	-	Ref.	Ref.
Opportunistic DFSA (other drugs ± alc.)					1.22 (0.53–2.84)	Idem
Proactive DFSA (involuntary substance consu.) ^d^					2.66 (0.73–9.70)	38.76 (12.15–123.59)^**^

^†^For variables satisfying the proportional odds assumption, the same odds ratio applies across both thresholds (shown as Idem).

^*a*^OR estimated using generalized ordinal logistic regression models with all covariates simultaneously.

^*b*^Less invasive assault: unwanted touching and/or kissing exclusively.

^*c*^More invasive assault: unwanted masturbation by another person, oral sex, and/or vaginal or anal penetration, with or without unwanted touching and/or kissing.

^*d*^Proactive DFSA included involuntary substance consumption through covert administration, force, coercion, or injection.

Differences in sample size across models reflect missing information in variables incorporated at each adjustment step, particularly perpetrator strategy.

Data are based on valid responses from participants who reported having experienced DFSA while partying.

^*^p < 0.05.

^**^p < 0.001.

#### 3.2.1 Sex of the victim

Women showed significantly higher odds of both any disclosure and disclosure to authorities compared with men across all models. After full adjustment, women had 1.74 times the odds of disclosure compared with men (Model 3: OR = 1.74; 95% CI: 1.06–2.87; *p* = 0.028; Table 4).

#### 3.2.2 Invasive nature of the sexual assault

Assault invasiveness was strongly associated with disclosure to authorities, but not with the likelihood of any disclosure vs. no disclosure. Victims of more invasive assaults had more than seven times the odds of disclosing the experience to authorities (Model 1: OR = 7.27; 95% CI: 3.18–16.65; p < 0.001). This association was slightly attenuated after adjustment for perpetrator characteristics (Model 2: OR = 5.55; 95% CI: 2.22–13.89; *p* < 0.001). After further adjustment for perpetrator strategy, the estimate changed direction and was no longer statistically significant (Model 3: OR = 0.52; 95% CI: 0.27–1.00; *p* = 0.051; Table 4).

#### 3.2.3 Perpetrator characteristics

Regarding perpetrator characteristics, assaults involving multiple perpetrators were associated with higher odds of both any disclosure and disclosure to authorities compared with assaults involving a single perpetrator (Model 2: OR = 3.34; 95% CI: 1.32–8.46; *p* = 0.011). Adjustment for perpetrator strategy eliminated the statistical significance of the association (Model 3: OR = 2.57; 95% CI: 0.87–7.62; *p* = 0.089; Table 4).

#### 3.2.4 Perpetrator strategy

Perpetrator strategy showed the strongest association with disclosure patterns. After full adjustment, victims of proactive DFSA involving involuntary substance consumption had substantially higher odds of disclosure to authorities than victims of opportunistic DFSA in which the perpetrator took advantage of the victim's voluntary substance consumption (Model 3: OR = 38.76; 95% CI: 12.15–123.59; *p* < 0.001). However, despite the large magnitude of the association, the wide confidence interval indicates considerable uncertainty regarding the precise effect size (Table 4).

Because disclosure to authorities was uncommon, particularly among participants reporting opportunistic alcohol-related DFSA, a sensitivity analysis was conducted. Within the Model 3 analytic sample (*n* = 429), 5 of 354 participants (1.4%) in the opportunistic alcohol-related DFSA group disclosed to authorities, compared with 2 of 43 (4.7%) in the opportunistic other-drug group and 11 of 32 (34.4%) in the proactive DFSA group. In a conventional binary logistic regression comparing disclosure to authorities with all other disclosure outcomes, proactive DFSA was associated with higher odds of disclosure to authorities than opportunistic alcohol-related DFSA (OR = 27.67, 95% CI 6.88–111.32; *p* < 0.001). In the Firth penalized logistic regression sensitivity analysis, the association was attenuated but remained strong and statistically significant (OR = 22.29, 95% CI 5.98–83.04; *p* < 0.001).

Perpetrator strategy was available for 455 of the 583 participants eligible for the disclosure analysis (78.0%), whereas 128 participants (22.0%) had missing information on this variable. The proportion of missing perpetrator-strategy data did not differ significantly by disclosure status: 18.6% among participants who disclosed to no one, 23.0% among those who disclosed only to their social environment, and 16.0% among those who disclosed to authorities (χ^2^(2) = 1.48, *p* = 0.477). When disclosure was dichotomized, missingness was 18.6% among participants reporting no disclosure and 22.7% among those reporting any disclosure (χ^2^(1) = 0.80, *p* = 0.371).

No statistically significant differences in missingness were observed according to sex, educational level, assault invasiveness, number of perpetrators, victim-perpetrator relationship, or age. In adjusted logistic regression, any disclosure was not associated with missing perpetrator-strategy data (aOR = 1.33, 95% CI 0.74–2.41; *p* = 0.342). Results were similar when the three disclosure categories were examined separately: compared with no disclosure, the adjusted odds of missing perpetrator-strategy data were 1.35 (95% CI 0.75–2.45; *p* = 0.318) for disclosure only to the social environment and 0.98 (95% CI 0.28–3.39; *p* = 0.977) for disclosure to authorities.

Assault invasiveness was significantly associated with perpetrator strategy (χ^2^(2) = 68.94, *p* < 0.001; Cramér's *V* = 0.40). Proactive DFSA accounted for 3.1% of less invasive assaults and 30.9% of more invasive assaults. Nevertheless, variance inflation factors were low (VIF = 1.30 for assault invasiveness and 1.26 for proactive DFSA perpetrator strategy; mean VIF = 1.15), providing no evidence of problematic multicollinearity.

## 4 Discussion

This study is the first in Spain to examine the prevalence and factors associated with disclosure patterns among young victims of DFSA occurring in party settings. The findings reveal a substantial hidden burden of victimization. 96 out of every 100 victims had never disclosed the assault to healthcare, police, or judicial authorities, whereas 18 out of every 100 had never disclosed the experience to anyone. This striking gap is particularly relevant given the high prevalence of DFSA in Spain, where approximately one in two women and one in four men have experienced DFSA while partying (10).

To our knowledge, no previous studies have examined DFSA disclosure in Spain; nevertheless, our findings are consistent with international research showing that disclosure of sexual assault occurs predominantly within informal social networks (12, 15). Informal support may provide valuable assistance; however, disclosure to formal institutions remains essential for accessing specialized healthcare and other victim-support services (13). Thus, the hidden burden of DFSA arises not only from complete non-disclosure, but also from disclosure remaining confined to informal social networks without reaching healthcare, police, or judicial authorities, thereby creating a critical bottleneck in the disclosure process. The observed discrepancy raises important questions regarding the role of informal support networks in shaping subsequent help-seeking pathways. Survivors' decisions to seek formal support are frequently preceded by disclosure to a close confidant (20, 29), and invalidating reactions discourage further disclosure and help-seeking through feelings of shame (30). Negative social reactions, including victim-blaming (31, 32), may stigmatize survivors and invalidate their experiences, ultimately functioning as a powerful silencing mechanism (33). Victim-blaming refers to the attribution of responsibility for a sexual assault to the victim rather than to the perpetrator. Such attributions are shaped by both observer characteristics and features of the assault, including rape myth acceptance, conformity to traditional gender roles, and victims' substance use; notably, intoxicated victims tend to be attributed greater responsibility than sober victims (34). Recent experimental evidence further indicates that victim-blaming and recognition of an incident as sexual assault are influenced by whether coercion involves physical force or alcohol-related incapacitation (35). These social judgements may be internalized by victims, reinforcing self-blame and concerns about being disbelieved and thereby discouraging further disclosure and help-seeking (17). Beyond its implications for survivors' access to support, non-disclosure also affects epidemiological surveillance by underestimating the magnitude of DFSA, thereby limiting the design, implementation, and evaluation of appropriate policy responses.

### 4.1 . Factors associated with disclosure

#### 4.1.1 Sex of the victim

Women were more likely than men to disclose the assault overall, although they tended to disclose it to their close social environment rather than to authorities. This pattern suggests that gender-related social norms may shape not only whether victims disclose, but also how they do so. Among women, gendered double standards surrounding alcohol and other drug use may reinforce self-blame and discourage formal reporting (34, 36). Previous research has shown that women's credibility as victims is often undermined when substance use is involved, with responsibility for the assault frequently shifted onto the victim (37–39). Among men, previous studies have reported a high prevalence of DFSA in party settings (10) which has been interpreted as reflecting a greater willingness to recognize victimization in contexts traditionally associated with masculinity, such as partying and heavy alcohol consumption (40, 41). However, recognizing victimization does not necessarily lead to disclosure. Persistent myths that question the legitimacy of male sexual victimization (42) and the enduring “female victim–male perpetrator” paradigm (43) may generate shame and isolation, discouraging disclosure despite victim recognition (44).

#### 4.1.2 Invasive nature of the sexual assault

More invasive assaults were less frequently disclosed within victims' close social environments, consistent with previous reviews (19). This may reflect greater shame and anticipated stigma, whereas disclosure to authorities was more frequent after more invasive assaults. These findings suggest that formal reporting may require victims to perceive the assault as exceeding a certain threshold of severity, despite evidence that less invasive sexual violence may also have important health consequences (45, 46), including in DFSA contexts (47, 48).

#### 4.1.3 Perpetrator characteristics

Overall disclosure was less frequent when the perpetrator was previously known to the victim, whereas assaults involving multiple perpetrators were more likely to be disclosed to authorities. These findings may reflect the influence of dominant social representations of sexual violence on disclosure decisions. Although most sexual assaults, including DFSA, are perpetrated by someone known to the victim and involve a single perpetrator (8, 49), the rape myth that sexual assaults are typically committed by strangers remains widespread (50–52). Similarly, although group-perpetrated DFSA accounts for fewer than 10% of cases in Spain (10), extensive media coverage of these incidents (53–55) may have made them more salient in the public imagination (9). Both assaults perpetrated by strangers and those involving multiple perpetrators may be perceived as more closely conforming to culturally stereotyped representations of “real rape” (52, 56) and, consequently, may be more readily recognized as sexual violence, potentially facilitating disclosure despite representing less common forms of DFSA.

#### 4.1.4 Perpetrator strategy

Perpetrator strategy emerged as the strongest factor associated with disclosure patterns. The introduction of perpetrator strategy in Model 3 substantially changed the association between assault invasiveness and disclosure to authorities. This change should not be interpreted as evidence of mediation, because the cross-sectional design does not establish the temporal ordering required for mediation analysis and perpetrator strategy is conceptually unlikely to be a consequence of assault invasiveness. Instead, perpetrator strategy may represent an antecedent or overlapping characteristic of the assault that could confound the observed association between invasiveness and formal disclosure. Although invasiveness and perpetrator strategy were moderately associated, multicollinearity diagnostics did not indicate problematic collinearity. Particularly noteworthy was the extremely low level of disclosure to authorities following opportunistic DFSA. By contrast, disclosure to authorities reached 34% among victims of proactive DFSA involving involuntary substance consumption. This finding is especially striking given that opportunistic DFSA accounts for the vast majority of DFSA occurring in party settings (8–10, 57). Thus, the most common form of DFSA may also be the least recognized and institutionally reported, with surveillance systems based on formal reporting capturing only a small fraction of its true population burden.

### 4.2 . Opportunistic DFSA as a “perfect storm”

The hidden burden associated with opportunistic DFSA may reflect the convergence of multiple interconnected barriers that should be considered jointly. These include gendered social stereotypes surrounding substance use (36) and DFSA-specific rape myths (58), particularly the widespread representation of DFSA as the covert administration of so-called “rape drugs” and the under-recognition of alcohol as a facilitating substance (9, 58, 59). Additional factors, such as substance-related memory impairment (60), the absence of physical injuries (61, 62), and delays in recognizing or disclosing the assault (63), may further contribute to uncertainty, self-blame, and anticipated stigma. Together, these mechanisms may reinforce the disclosure bottleneck identified in the present study (33, 44, 49).

#### 4.2.1 Memory impairment

Psychoactive substances frequently impair victims' ability to reconstruct the assault and increase uncertainty about what occurred. Memory loss and fragmented recollections are among the most common manifestations of DFSA (60, 64) and may lead victims to question their own memories and anticipate disbelief from others, discouraging disclosure to healthcare, police, or judicial authorities (63).

#### 4.2.2 Lack of visible injuries

Compared with assaults involving overt physical force, incapacitated victims typically offer little or no resistance, reducing the likelihood of sustaining physical or genital injuries (62, 65, 66). Accordingly, studies from different countries have consistently reported a lack of genital and extragenital injuries among many victims (21, 67, 68). In the absence of visible physical evidence, survivors may minimize the assault, question whether a crime has occurred, or anticipate disbelief from others, thereby hindering recognition of DFSA as a criminal offense warranting formal reporting (49, 63).

#### 4.2.3 Delayed recognition and help-seeking

Disclosure is often delayed following opportunistic DFSA, as DFSA-specific rape myths may hinder recognition of victimization and subsequent disclosure. This is consistent with forensic evidence showing longer delays to presentation among suspected DFSA cases than among other sexual assault victims (57, 66, 69).

Taken together, these mechanisms may create a “perfect storm” for under-recognition and under-disclosure, with implications for help-seeking, access to formal support, recovery, and criminal justice. Many characteristics of opportunistic DFSA that hinder disclosure have also been identified as barriers within the criminal justice system. Alcohol intoxication, inconsistencies in the victim's account, and lack of physical evidence may reduce police investigative engagement, increase skepticism toward the victim's account (70, 71), and weaken the perceived strength of the case during prosecution (49). These factors may contribute to the exceptionally low levels of disclosure to authorities observed in the present study. Despite these barriers, Spanish judicial rulings show that opportunistic assaults are the predominant DFSA cases reaching the courts (72), suggesting that these cases represent only the visible fraction of a much larger hidden burden of victimization. Figure 1 summarizes these conceptual interactions as a reinforcing cycle in which barriers to recognition and disclosure may limit access to formal support and contribute to the continued hidden burden of opportunistic DFSA.

**Figure 1:**
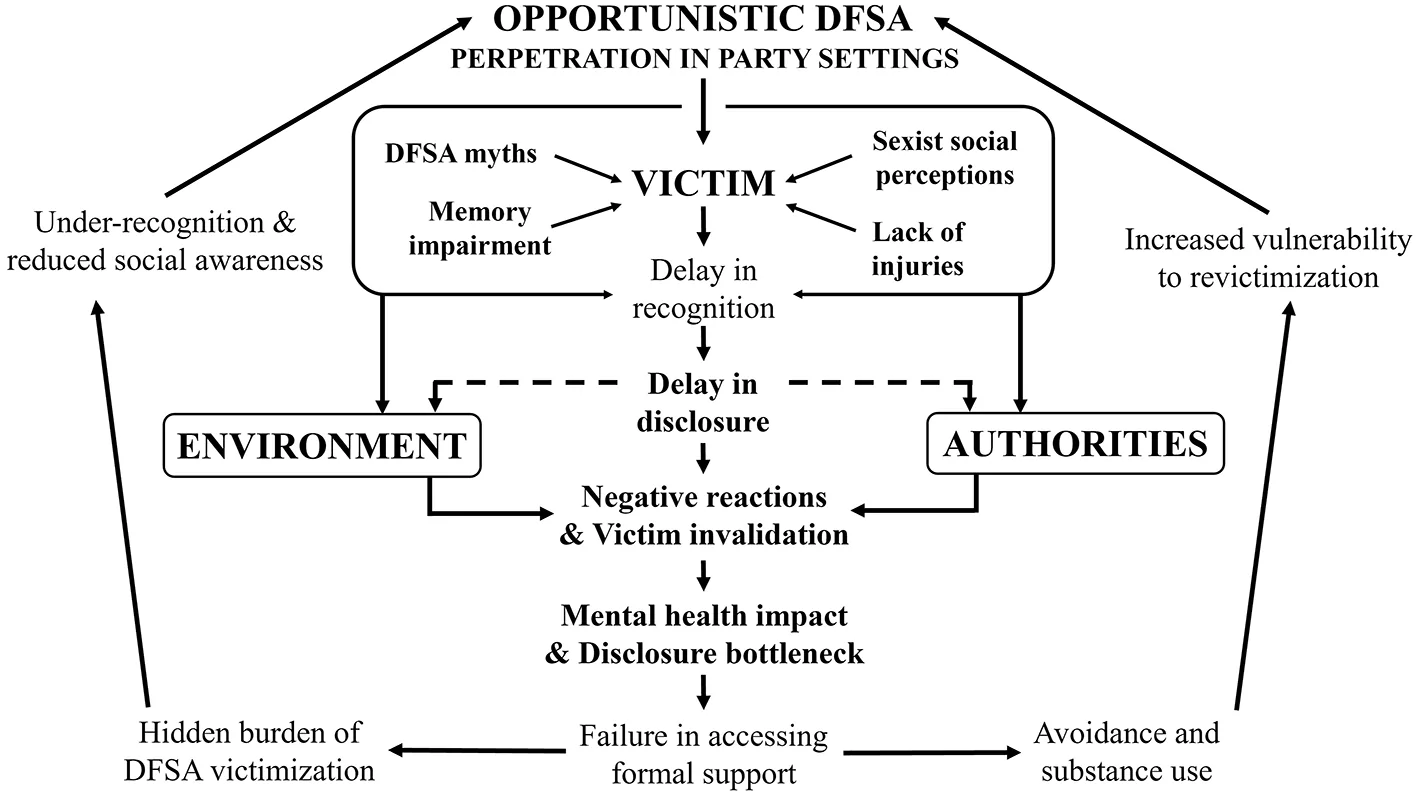
The opportunistic DFSA disclosure paradox: a “perfect storm” of interconnected barriers to recognition, disclosure, help-seeking, and recovery. (Figure created by the authors.)

### 4.3 . Aligning DFSA prevention with epidemiological evidence

The magnitude of the hidden burden of DFSA victimization underscores the need to raise public awareness of the realities of DFSA in party settings, particularly the barriers that prevent victims from recognizing and disclosing their experiences. These findings also call into question the emphasis of current prevention campaigns, as public messages may inadvertently shape social representations of sexual violence (73, 74). Because proactive DFSA is more readily recognized, victims of opportunistic DFSA may interpret fragmented memories through the dominant narrative of covert drink spiking. Rather than reflecting intentional misrepresentation, this process may arise from the interaction between impaired memory and prevailing social representations when making sense of highly ambiguous experiences.

Campaigns that portray DFSA primarily as covert drink spiking—including those promoting drink-spiking detection technologies—may therefore reinforce DFSA-specific rape myths while obscuring opportunistic DFSA (75), the most common form in party settings. Prevention efforts should instead reflect the epidemiological reality of DFSA by addressing both covert substance administration and assaults facilitated by voluntary alcohol or other drug use. Importantly, this broader approach must avoid shifting responsibility onto victims who use substances and instead emphasize perpetrators' exploitation of intoxication or impaired capacity to consent.

Prevention should also address the consequences of negative social reactions following disclosure, which may not only discourage help-seeking but also reinforce avoidant coping (18, 76–78). For some survivors, alcohol or other drug use may become a maladaptive strategy to manage post-traumatic distress (12, 78). Other avoidant responses may involve compulsive sexual behavior and problematic pornography use (79), which may serve as coping mechanisms for trauma-related distress among some survivors (80–83). Although evidence remains limited, such behaviors may form part of patterns associated with subsequent vulnerability to opportunistic DFSA. Pornography use has been linked to greater rape myth endorsement (84) and greater tolerance of unwanted sex (85), while its combination with alcohol has been associated with increased victim blaming, greater acceptance of sexual victimization (86, 87), and substantially higher odds of self-reported sexual victimization (88). This potential pathway warrants further investigation in longitudinal research on revictimization following opportunistic DFSA.

These findings also suggest several priorities for future research and practice. Reducing the bottleneck between informal disclosure and access to formal support may require measures that strengthen the confidence of victims of opportunistic DFSA in healthcare, law-enforcement, and other formal institutions. This may require not only specific professional training and protocols, but also an active and visible role for healthcare and law-enforcement institutions and professionals in publicly recognizing opportunistic DFSA, raising awareness of its realities, and reinforcing the legitimacy of victims' experiences. From a clinical perspective, longitudinal research should examine whether maladaptive coping responses following sexual victimization are associated with increased vulnerability to subsequent opportunistic DFSA, and whether such mechanisms contribute to cycles of revictimization, without implying any responsibility on the part of victims. Legal and forensic research should examine whether victims of opportunistic DFSA may report their experiences in ways that more closely conform to the socially dominant narrative of proactive DFSA, particularly covert or involuntary drug administration. From a legal and public health perspective, studies should compare DFSA prevalence with reporting, prosecution, and conviction rates in Spain and examine criminal justice attrition in proactive and opportunistic DFSA to determine whether, and at which stages, opportunistic cases are disproportionately lost.

By exposing the hidden burden of DFSA and the barriers to formal support, these findings contribute to Sustainable Development Goals concerning health, gender equality, and access to justice, while supporting evidence-based, trauma-informed responses aligned with DFSA epidemiology.

### 4.4 . Limitations and strengths

Several limitations should be considered. The cross-sectional design precludes causal inference, and the retrospective self-report of highly sensitive experiences may be affected by recall bias, social desirability bias, or reluctance to disclose sexual victimization, despite the anonymous nature of the survey. Because assault characteristics and disclosure were assessed only for the most recent DFSA episode, cumulative victimization history could not be considered. Previous DFSA experiences may have influenced recognition and disclosure of the most recent assault. Although quota sampling approximated the distribution of the target population and the response rate was 93.8%, participation was voluntary and based on an online panel, which may have introduced self-selection bias. Sociodemographic information was collected selectively to preserve respondents' comfort and sincerity, and the limited representation of sexual-minority participants and the absence of a measure of gender identity restricted more detailed analyses of sexual and gender diversity. Another important limitation is the reduced sample size in some multivariable models, particularly Model 3, which reflected missing information in assault-related variables, most notably perpetrator strategy. Additional analyses showed no significant association between missingness in perpetrator strategy and disclosure status, either in unadjusted comparisons or after adjustment for the principal observed sociodemographic and assault-related characteristics. Missingness was also not significantly associated with sex, age, educational level, assault invasiveness, number of perpetrators, or victim–perpetrator relationship. These findings provide some reassurance against substantial differential missingness according to observed characteristics. Nevertheless, missingness related to unmeasured characteristics—such as recall of the assault circumstances, intoxication-related memory impairment, or uncertainty regarding how the assault was facilitated—cannot be excluded. Accordingly, estimates involving perpetrator strategy should be interpreted with appropriate caution. Despite these limitations, the large national general-population sample allowed us to capture DFSA experiences that remain outside formal support systems. By integrating these findings with a conceptual interpretation of disclosure barriers, this study provides novel evidence on the invisibility of opportunistic DFSA and its implications for research, prevention, and victim support.

### 4.5 . Conclusions

The hidden burden of DFSA victimization in party settings is substantial, with disclosure to healthcare, police, or judicial authorities remaining exceptionally rare despite the high prevalence of these assaults. Disclosure is largely confined to survivors' close social networks, creating a major bottleneck in access to formal support. Opportunistic DFSA, the most common perpetrator strategy in party settings, showed the lowest levels of formal disclosure. Given the small number of formal disclosures, associations involving this outcome should nevertheless be interpreted with caution. This pattern may be related to the combined influence of DFSA-specific rape myths, victim-blaming attitudes, substance-related memory impairment, lack of visible injuries, and delayed recognition of the experience as sexual violence. Limited access to professional support may hinder timely care and recovery. Prevention campaigns should avoid portraying DFSA primarily as covert drink spiking and should explicitly address opportunistic assaults facilitated by voluntary alcohol or other drug use, while ensuring that such messaging does not attribute blame to victims and clearly places responsibility on perpetrators. Improving public recognition of opportunistic DFSA, reducing victim blaming, strengthening institutional responses, and facilitating timely, trauma-informed support should therefore be key priorities for prevention and response.

## Data Availability

The data analyzed in this study is subject to the following licenses/restrictions: Dataset is not public. Requests to access these datasets should be directed to lsordo@ucm.es.
