# Supplementary material for: Breaking the silence on drug-facilitated sexual assault: disclosure patterns and barriers to reporting among young people in Spain
Source: Front Public Health. 2026 Oct 19;14:1924643. doi: 10.3389/fpubh.2026.1924643 (PMC13724494; doi:10.3389/fpubh.2026.1924643)
Supplement: Supplementary file 1 [file Supplementary_File_1.docx]

**Multimedia Appendix 1. Expanded extract of the questionnaire relevant to DFSA victimization and disclosure (English translation).**

*The original questionnaire covered additional topics beyond the scope of the present study. The following expanded extract includes the items used to identify DFSA victimization, characterize the most recent DFSA episode, measure disclosure, and derive the sociodemographic and assault-related variables included in the present analyses. Items not relevant to the present study have been omitted.*

**Participation Note:** Participation is completely voluntary and anonymous. All shared data are subject to the Spanish Organic Law 3/2018, of December 5, on Personal Data Protection and Guarantee of Digital Rights. We guarantee the absolute anonymity and confidentiality of your responses, in strict compliance with the laws on statistical secrecy and personal data protection.

It is very important that you answer honestly. Please remember that everything you say is completely confidential. Once the information is recorded anonymously, individual questionnaires are destroyed.

Some questions address intimate aspects of sexual life and experiences, so we thank you in advance for your honest answers. Thank you for participating in our survey.

**Do you agree to participate in this survey?**

- Yes
- No

**Next, we will ask you about your sexual experiences in the past. It is very important that you answer honestly. Everything you say is completely anonymous and confidential.**

**Identification of DFSA victimization.**

**Have you ever in your life, while partying or immediately after, experienced any of the following episodes without your consent while under the influence of alcohol or other drugs?**

| **Behavior** | **Yes** | **No** | **Prefer not to answer** |
| --- | --- | --- | --- |
| Kissing |  |  |  |
| Touching |  |  |  |
| Masturbation |  |  |  |
| Oral sex (fellatio, cunnilingus) |  |  |  |
| Vaginal penetration |  |  |  |
| Anal penetration |  |  |  |

***Routing note:*** *Participants who reported at least one non-consensual sexual experience under the influence of alcohol or other drugs were subsequently asked to answer the following questions with reference to the most recent episode. For clarity, the original order of the questionnaire items has been rearranged in this appendix to group related questions by analytical domain; therefore, the order presented here does not necessarily reflect the order in which the items were administered to participants.*

***Analytical note:*** *For the present analyses, unwanted kissing and/or touching only were classified as less invasive assaults, whereas masturbation by another person, oral sex, and/or vaginal or anal penetration were classified as more invasive assaults.*

**Characteristics of the most recent DFSA episode.**

**Thinking about the most recent time this happened, was the act carried out by…?**

- One man
- One woman
- A group of men
- A group of women
- A group of men and women
- I don’t know
- Prefer not to answer

**Thinking about the most recent time this happened, how would you describe your relationship with the person or persons who carried out the act at that time?**

- Stranger(s)
- Friend(s)
- Classmate(s) or co-worker(s)
- Family member(s)
- Current partner
- Former partner
- Acquaintance(s)
- Someone I had just met / people I had just met
- I do not know who the perpetrator(s) were
- Prefer not to answer
- Other (please specify): __________

***Analytical note:*** *For the present analyses, responses were used to derive perpetrator sex, number of perpetrators (one vs. multiple), and previous relationship with the perpetrator (known vs. unknown).*

**Thinking about the most recent time this happened, under the effects of which substances were you, and how were those substances consumed? You** may **select more than one response in each row.**

| **Substance** | **I consumed this substance voluntarily** | **Someone made me consume it without my knowledge** | **Someone forced me to consume it or pressured me into consuming it** | **Someone injected the substance into me by needle** | **I did not consume this substance** | **I do not know whether I consumed it** | **Prefer not to answer** |
| --- | --- | --- | --- | --- | --- | --- | --- |
| Alcohol | ☐ | ☐ | ☐ | ☐ | ○ | ○ | ○ |
| Cannabis (marijuana, hashish) | ☐ | ☐ | ☐ | ☐ | ○ | ○ | ○ |
| Cocaine | ☐ | ☐ | ☐ | ☐ | ○ | ○ | ○ |
| Amphetamines (MDMA, EME, ecstasy, crystal, speed, mephedrone, cathinones, methamphetamine) | ☐ | ☐ | ☐ | ☐ | ○ | ○ | ○ |
| Hallucinogens (LSD, mushrooms, etc.) | ☐ | ☐ | ☐ | ☐ | ○ | ○ | ○ |
| Tranquilizing, sedative, or anxiolytic medications | ☐ | ☐ | ☐ | ☐ | ○ | ○ | ○ |
| GHB (liquid ecstasy) | ☐ | ☐ | ☐ | ☐ | ○ | ○ | ○ |
| Chloroethane | ☐ | ☐ | ☐ | ☐ | ○ | ○ | ○ |
| Poppers | ☐ | ☐ | ☐ | ☐ | ○ | ○ | ○ |
| Ketamine | ☐ | ☐ | ☐ | ☐ | ○ | ○ | ○ |
| Burundanga (scopolamine) | ☐ | ☐ | ☐ | ☐ | ○ | ○ | ○ |
| Laughing gas / nitrous oxide balloons | ☐ | ☐ | ☐ | ☐ | ○ | ○ | ○ |
| Some unknown or undetermined substance | ☐ | ☐ | ☐ | ☐ | ○ | ○ | ○ |
| Another substance not mentioned above | ☐ | ☐ | ☐ | ☐ | ○ | ○ | ○ |

***Analytical note:*** *F*or the present analyses, perpetrator strategy was classified as: (1) opportunistic DFSA involving voluntary alcohol consumption only; (2) opportunistic DFSA involving voluntary consumption of other drugs, with or without alcohol; or (3) proactive DFSA involving involuntary substance consumption through covert administration, force, coercion, or injection.

**Disclosure.**

**Thinking about the most recent time this happened, who did you tell about what happened? Select as many options as necessary.**

- Friends
- Relatives
- Partner or ex-partner
- School or educational center (teacher, management staff, counseling services)
- Someone from work (boss, co-worker, employee, etc.)
- Medical or psychological services (doctor, nurse, psychologist)
- Police / Court (National Police, Guardia Civil, Local Police)
- I called a victim helpline or support service
- I shared it on social media (Twitter, Instagram, TikTok, etc.)
- Lawyer
- I did not tell anyone
- Prefer not to answer
- Other (specify)

***Analytical note:*** *F*or the present analyses, responses were grouped into three mutually exclusive disclosure categories: (1) no disclosure; (2) disclosure to the close social environment only; and (3) disclosure to authorities, with or without previous disclosure to the close social environment. Disclosure to authorities included medical or psychological services and police or judicial institutions. All other disclosure recipients were classified within the close social environment.

**Sociodemographic characteristics.**

**Indicate your sex as it appears on your ID card, passport, or a similar official document:**

- Man
- Woman
- Prefer not to answer

**How old were you on your last birthday? Enter your age in digits:**

**From the following list, which option best describes your sexual orientation?** By sexual orientation, we mean which sex you are attracted to.

- Heterosexual (people of the opposite sex)
- Homosexual (people of your same sex)
- Bisexual (people of both sexes)
- Other sexual orientation
- I haven’t decided yet / I don’t know
- Prefer not to answer

**When discussing politics, the terms left and right are commonly used. On a scale from 1 to 10, where 1 means "far left" and 10 means "far right," where would you place yourself on this scale?**

**Regarding nationality, your nationality is:**

- Spanish
- Spanish and another nationality
- Another nationality
- Prefer not to answer

**Currently, among all the members of your household living with you, including yourself, approximately, what is your total monthly net income?**

- No monthly income
- Less than or equal to €1,000
- Between €1,001 and €2,000
- Between €2,001 and €3,000
- Between €3,001 and €4,000
- More than €4,000
- I don’t know
- Prefer not to answer

**What is the highest level of education you have completed?**

1. Less than primary education
2. Primary education
3. Secondary education
4. Basic vocational training
5. Intermediate vocational training
6. High school / Baccalaureate
7. Higher vocational training
8. University studies (Bachelor’s degree, Licentiate, etc.)
9. Postgraduate studies (Master’s, Postgraduate, Doctorate, etc.)
10. Prefer not to answer
